# Integrated Care Management to Improve Diabetes Outcomes in Refugee and Immigrant Patients (I-Care)

**DOI:** 10.1089/heq.2020.0143

**Published:** 2021-11-17

**Authors:** Waseem Sous, Christina D. Lupone, Megan A Harris, Ayan Mohamed, Liban Mohamed, Mary Jo Lakomski, Simone Seward, Andrea V. Shaw

**Affiliations:** ^1^ Department of Internal Medicine, SUNY Upstate Medical University, Syracuse, New York, USA.; ^2^ Department of Public Health and Preventive Medicine, and SUNY Upstate Medical University, Syracuse, New York, USA.; ^3^ SUNY Upstate Medical University, Institute for Global Health and Translational Science, Syracuse, New York, USA.; ^4^ SUNY Upstate Medical University College of Medicine MD/MPH Program, Syracuse, New York, USA.; ^5^ Department of Department of Pharmacy, SUNY Upstate Medical University, Syracuse, New York, USA.

**Keywords:** community-based organizations, Certified Diabetes Care and Education Specialist, chronic disease management, refugee, social determinants of health

## Abstract

**Purpose:** Refugee and immigrant patients face significant barriers to health care and are more likely to have poorly controlled chronic disease than the general U.S. population. I-Care aims to improve health equity for refugees and immigrants who face a disproportionate burden of chronic disease.

**Methods:** Refugees and immigrants with uncontrolled diabetes and associated cardiovascular risk factors were enrolled in a care management program within an academic adult medicine clinic. The program utilized a care manager to coordinate care and services between designated primary care providers, affiliated clinical teams, and community partners. Health literacy, chronic disease parameters, and care utilization were assessed at enrollment and 8–12 months later.

**Results:** A total of 50 refugees and immigrants were followed for 8 to 12 months. Clinical parameters found a reduced mean HbA1c from 9.32 to 8.60 (*p*=0.05) and reduced low-density lipoprotein mean from 96.22 to 86.60 (*p*=0.01). The frequency of normal blood pressures was 9 (18%) at enrollment and 16 (32%) at 1 year. The cumulative frequency of emergency room visits decreased from 66% to 36% and hospitalizations from 22% to 8%. Rates of comprehensive care monitoring, including monofilament testing and one-time ophthalmology visits, increased from 60% to 82% and from 32% to 42%, respectively. Cumulative frequency of interdisciplinary support engagement with pharmacy and nutrition visits increased from 58% to 78% and from 26% to 38%, respectively.

**Conclusion:** This program highlights the importance of a multidisciplinary community-engaged care model that has demonstrated improvement in quality metrics and health care costs for refugees and immigrants.

## Introduction

Refugees and immigrants have specific health care needs, as well as a unique set of barriers to accessing care, including language, education, health literacy, and transportation, which have been exacerbated by the COVID-19 pandemic.^1–6^ In Onondaga County, located in central New York (NY), 7.6% of residents are foreign-born and this rate increases to 12.5% in Syracuse, where there are many recently resettled refugees.^7^ Since 2000, >10,000 refugees have resettled in the Syracuse area.^8^

The Adult Medicine Clinic at State University of New York (SUNY) Upstate Medical University, located in Syracuse, NY, provides primary care to 900 refugee and immigrant patients annually, who speak over 40 languages. Of these patients, over 80% reside within the city of Syracuse. The rate of diabetes in this population is 20% compared with 10.2% in the general Onondaga county population.^9^ Of the refugee and immigrant patients who had an HbA1c in the last year, 16% had an HbA1c >8%. Of the 900 refugees seen by Adult Medicine, almost 25% have had at least one blood pressure (BP) reading >130/80. Research shows that refugees are at increased risk for chronic disease^10,11^ and are less likely to meet diabetes and BP management goals than the general U.S. population.^12,13^

It has previously been shown that new immigrants enter the United States with limited financial resources, often lack health insurance, and demonstrate difficulty in navigating the health care system due to limited English proficiency, resulting in decreased health care utilization and delays in treatments.^14^ The average refugee family resettled to Syracuse spent a decade in a refugee camp before coming to Syracuse. Poverty impacts a significant number of social determinants of health (SDOH), including access to safe housing, transportation, employment opportunities, healthy foods, physical activity, and educational background. These nonmedical needs have a large influence on an individual's health outcomes, and research shows that SDOH play a substantial role in most health inequalities.^15^

Ninety percent (90%) of preventive care visits for diabetes are delivered by primary care providers (PCPs) with a focus on episodic treatment.^16^ Effective, culturally appropriate primary care requires understanding the context of the refugee experience and its physical and emotional sequelae; addressing geographic, linguistic, economic, and cultural barriers; and providing high-quality care within the context of the traditional family structure, gender roles, cultural norms, and social support systems.^17^ Many PCPs have inadequate training in refugee health care, which poses a significant barrier to addressing complex SDOH and medical needs within the confines of a traditional clinic visit.^18^

I-Care was developed at Upstate to improve health equity for refugees and immigrants who face a disproportionate burden of chronic disease and many barriers to accessing and navigating a complex health care system. I-Care's approach includes the following: (1) comprehensive person-centered care provided by a multidisciplinary team coordinated by a designated care manager to support refugee and immigrant patients; and (2) collaboration with community-based organizations (CBOs) working with refugee and immigrant populations to train and support culturally and linguistically aligned community health navigators in patient outreach and advocacy.

## Methods

I-Care is a grant funded patient care program aimed to improve outcomes for diabetic patients in the refugee and immigrant community in Syracuse, NY. This study serves as an evaluation of the funded program to assess whether the clinical goals were met. It was reviewed by the SUNY Institutional Review Board (IRB) (Project 1477069-1) and determined to not meet the definition of human subjects research under the purview of the IRB according to federal regulations.

### The I-Care program

**FIG. 1.**
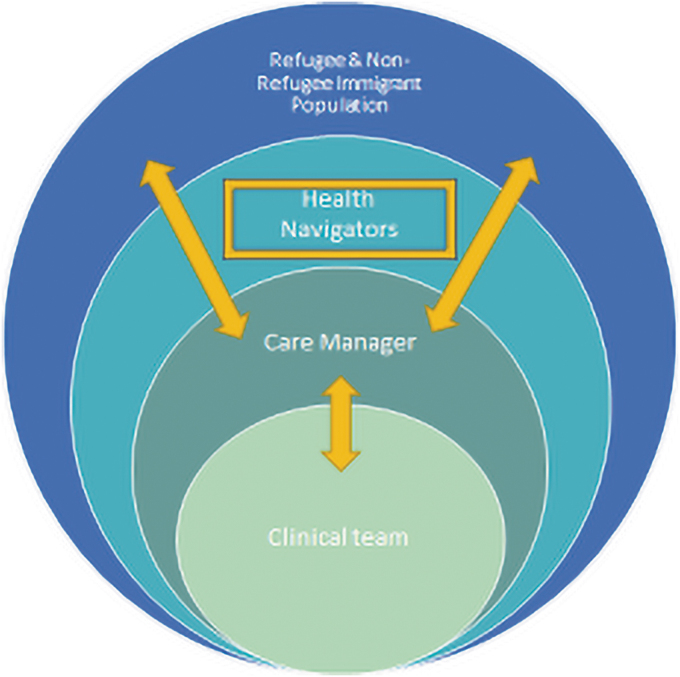
I-Care model for refugee and immigrant population. Engaged care manager functions as a liaison between the clinical team and community-based health navigator team to facilitate ongoing communication of care plans and care needs for culturally and linguistically diverse patients.

SUNY Upstate's Adult Medicine Clinic is an academic teaching clinic in the urban setting of Syracuse, NY, serving as a medical home for nearly 3400 patients, 26% non-English speaking and 76% insured through Medicaid or Medicare. From this clinic population, refugee and immigrant adult patients (ages >18 years) with poorly controlled diabetes (HbA1c >8%) and associated cardiovascular risk factors (BP >130/80, elevated low-density lipoprotein [LDL] >100) were offered enrollment in the I-Care program. Patients were identified through referrals made to our clinical pharmacist team and reports generated through patient-centered medical home to identify patients with uncontrolled diabetes metrics who had a designated language preference other than English and social documentation confirming they were foreign-born. Patients were enrolled from November 2019 to August 2020. Patients were offered enrollment with a care manager who served as a liaison between the clinical team, CBOs, and the patients to assist with coordination of care and services (Fig. 1). Interested patients were offered connection to a culturally and linguistically congruent community health navigator hired by one of two CBOs supporting refugee and immigrant communities. These CBOs include the Catholic Charities of Onondaga County (CCOC) and Refugee and Immigrant Self-Empowerment (RISE).

The I-Care multidisciplinary team held regular meetings to develop the administrative processes required to implement I-Care including workflows and referral processes; develop culturally and linguistically sensitive outreach and recruitment materials for CBOs; establish evaluation reporting structures; and execute contracts. The clinical team from within Upstate Adult Medicine consisted of an administrative lead, a care manager, a pharmacist, a registered dietician, a certified diabetes care and education specialist, and a PCP. Engagement with CBOs involved contracted time to support health navigators in their training around confidentiality, patient navigation, diabetes education, and community outreach. Health navigators met with the clinical team bimonthly to review cases and collective challenges in navigating care.

### Outcome measures: SDOH and chronic disease

Health literacy was assessed utilizing the Refugee Health Literacy Assessment Tool (R-HLAT)—a tool developed by this team for chronic disease health literacy assessment in refugee populations of diverse backgrounds and countries of origin.^19^ Based on health literacy screening results, an in-depth interview with the care manager addressed the needs and challenges for each patient. A person-centered plan of care was developed in partnership with the patient and was reflective of the needs and assets required to support chronic disease management and overall health. The care manager served as the liaison between the medical team, associated referrals, and the CBO health navigator.

The care manager was an integral part of the care team who met patients alongside primary care, pharmacy, and nutrition visits and engaged in discharge planning and follow-up needs. Routine primary care visits occurred quarterly, but additional visits were arranged based on individual patient needs. Regular patient outreach was performed in a manner that aligned with individual patient needs (i.e., calls with an interpreter, emails, texts, and/or office, home, or community visits).

### CBOs and health navigators

CBOs identified 8 health navigators, collectively speaking 19 languages, to engage and support chronic disease management for patients interested in further community support around chronic disease management. The I-Care team (including a pharmacist, a nutritionist, and medical providers) developed training for health navigators around patient confidentiality, patient navigation, and diabetes management. Diabetes management training covered disease process, nutritional management, medication management, and disease monitoring. Training took place over formal half-day sessions. Continued interface between health navigators and the care team occurred biweekly to address concerns for specific patients' needs.

The extent of health navigator involvement with an individual patient was driven by an individualized care plan. Examples of health navigator activities included the following: assisting patients in selecting culturally appropriate foods that align with a diabetic diet; accompanying patients to appointments; helping patients navigate benefit programs; participating in physical activities with patients; and raising awareness of preventive health issues.

### Data collection and statistical analyses

The R-HLAT and SDOH parameters were reviewed and recorded near the time of enrollment for each patient. Clinical outcome measures were reviewed via chart review in Upstate's EPIC electronic health record at the time of enrollment (i.e., most recent clinical parameters at the time of enrollment and care access parameters over the prior 12 months), as well as 8–12 months postenrollment. Clinical parameters included HbA1c, BP, LDL levels, urine microalbumin levels, as well as monofilament foot examination and dilated eye examination findings. Care utilization was assessed by frequency of patient visits with the primary care, ophthalmology, nutrition, and pharmacy teams, as well as by history of ED visits and hospitalizations in the 12 months before enrollment.

Reviewed data were transferred to this program's REDCap project by trained study personnel. REDCap is a secure, HIPAA-compliant web application used extensively at Upstate for data storage and management.^20,21^

Data analyses included calculation of frequencies and means. Paired sample *t*-tests were conducted to compare participant data at enrollment and up to 1 year postenrollment. Results were considered significant if *p*≤0.05. SPSS Statistics Version 27.0 was used for all statistical analyses.

## Results

### Sociodemographic characteristics

The median age of I-Care participants was 48 years (range 20–89 years) and 30 (60%) of the participants were female. Participants reported 11 different countries of origin and 15 different countries of residence before arrival to the United States, with Somalia being the most frequent country of origin (40%) and Kenya being the most frequent country of residence before U.S. arrival (22%). Year of U.S. arrival ranged from 2004 to 2019, with 2010 as the most frequently reported (16%). Somali was the most common (32%) of the 13 different primary spoken languages reported among the cohort.

### Diabetes and cardiovascular risk management

Table 1 displays the mean values and paired *t*-test statistics of clinical outcome measures. The mean HbA1c and LDL were significantly decreased at 1 year postenrollment (*p*=0.05 and *p*=0.01, respectively). Monofilament testing completion increased from 30 (60%) to 41 (82%).

**Table 1.** Clinical Outcome Measures at Enrollment and 1-Year Postenrollment

	Enrollment		1-Year post		Paired T-test		
	Mean	SD	Mean	SD	T	DF	*p*
Urine microalbumin	100.60	247.07	77.54	146.41	1.12	47	0.27
Hemoglobin A1C	9.32	2.33	8.60	2.15	1.97	49	0.05
LDL	96.22	38.38	85.60	35.16	2.78	49	0.01
Body mass index	30.76	8.44	30.75	8.87	0.32	49	0.96

HbA1c >14 and urine microalbumin <12 were approximated to reflect a value of 14 and 12, respectively. Urine microalbumin was unavailable for two individuals.

DF, degree of freedom; LDL, low-density lipoprotein; SD, standard deviation.

**FIG. 2.**
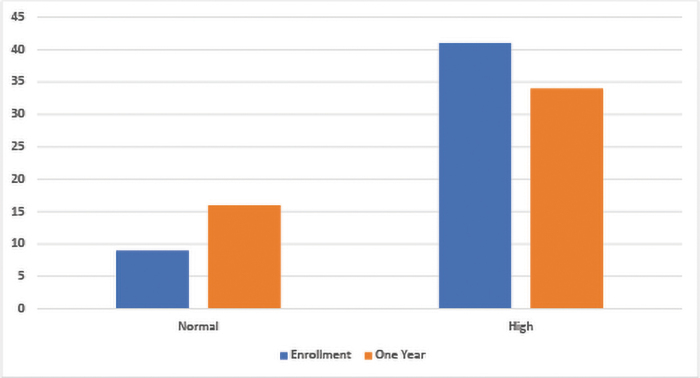
Frequency of blood pressure measurements at enrollment and 1-year postenrollment to I-Care. Normal includes elevated measurements according to the 2017 American College of Cardiology/American Heart Association Guidelines.

The frequency of BPs within normal limits was 9 (18%) at enrollment compared with 16 (32%) at 1 year postenrollment. The rates of elevated and high BPs decreased from 18 (36%) to 14 (28%) and from 23 (46%) to 20 (40%), respectively. Figure 2 illustrates the frequency of normal and high BP measurements. The 2017 American College of Cardiology/American Heart Association Guideline for the Prevention, Detection, Evaluation, and Management of High Blood Pressure in Adults was used as a reference for BP parameters.^22^

### Health care utilization

The cumulative frequency of emergency room visits decreased from 80 (66%) in the year before I-Care enrollment to 42 (36%) during the year engaged in I-Care. Total hospitalizations decreased from 11 (22%) in the year before I-Care enrollment to 4 (8%) during the year engaged in I-Care. The cumulative frequency of one-time ED visits decreased from 18 (36%) to 9 (18%) and one-time hospitalization from 9 (18%) to 3 (6%). One-day procedures and surgeries (e.g., heart catheterization for two individuals and septoplasty and elective circumcision for two others) were not included as hospital admissions.

**FIG. 3.**
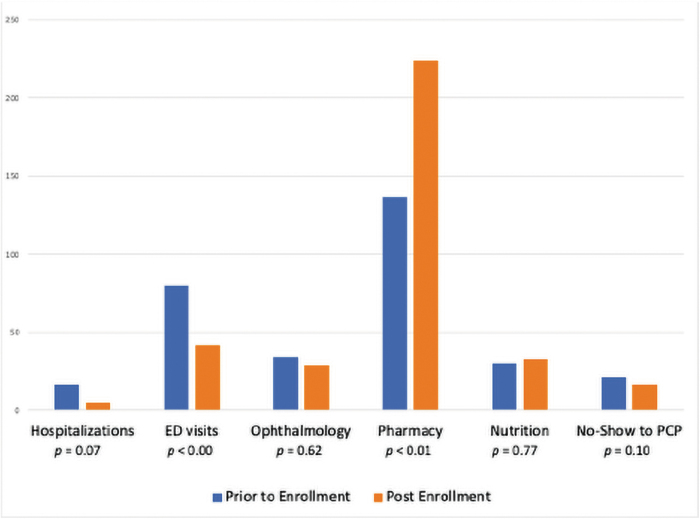
Frequency of visits 1 year before enrollment and 8–12 months postenrollment to I-Care. Paired *t*-test statistics reported for mean of total visits per group.

The cumulative frequency of ophthalmology visits decreased from 34 (44%) at enrollment to 29 (48%) at 1 year postenrollment, with the rate of one-time visits increasing from 16 (32%) to 21 (42%). The cumulative frequency of pharmacy and nutrition visits increased from 137 (58%) to 224 (78%) and from 30 (26%) to 33 (38%), respectively. See Figure 3 for frequency of clinical visits, including PCP no-shows.

### Health literacy and SDOH

The R-HLAT facilitated assessment of individuals' health literacy in four categories: medication, insurance and preventative care, appointments, and self-advocacy—highlighting areas of vulnerability for this population. For example, 36% of participants reported that they did not know what to do if they had questions about their medications, 42% reported that they did not know what to do if told their health insurance is not active or not working, and 38% reported not knowing how to schedule or change an appointment. Nearly all participants (98%) reported at least one medication barrier, with “difficulty picking up medications from the pharmacy” (64%) and “language barriers” (44%) as two of the most frequently reported barriers.

Regarding living situations, 6 (12%) participants reported living alone, 33 (66%) reported living with children, and 25 (50%) reported living with spouses. Regarding social support systems, 28 (56%) reported family, 18 (36%) reported children, 6 (12%) reported friends, 9 (18%) reported spouse, 1 (2%) reported case manager, and 4 (8%) reported no social support system. The most commonly reported sources of income among the cohort included public assistance (44%), employer (26%), and Supplemental Security Income (SSI) (26%). The most commonly reported means of transportation to medical appointments was family members (44%). Twenty-five (50%) participants reported no formal education, 14 (28%) reported completion of primary school, and 8 (16%) reported completion of secondary school. All participants had health insurance coverage—98% received coverage through Medicare or Medicaid health plans, and 1 participant (2%) had a private health insurance plan.

### CBO engagement and interventions

Three health navigator training sessions were organized to support diabetes education. Participants unanimously reported increased knowledge about diabetes care and increased comfort with utilization of pharmacy and nutritional resources. Regular feedback sessions between health navigators and the clinical team addressed common themes and challenges around chronic disease management among refugees and immigrants, including fear of the diagnosis of diabetes, mistrust of health care providers, and misconceptions related to chronic diabetes management. These themes were incorporated into individualized care plans and discussed during biweekly meetings between community health navigators, the care manager, and the clinical team.

## Discussion

There are numerous social and structural barriers to achieving health equity for refugee and immigrant populations in the United States. These include language barriers, inadequacies in cross-cultural communication, high level of trauma-based experiences that compound mental and physical health, varied educational backgrounds, limited health literacy, and discrimination in both the community and health care setting.^23^ The I-Care program is a multidisciplinary care model that engages a care manager as a liaison between a medical team and a network of community health navigators to address the disproportionate burden of uncontrolled chronic disease and barriers to care for a vulnerable patient population. This clinical model has established a trusted line of communication and means to care access through a Somali-speaking care manager, who is a former resettled refugee to Syracuse. Over the past 1 year, the care team has grown in its ability to provide culturally competent care and meet the needs of a diverse patient population. The trust, confidence, and trajectory of improved clinical outcomes seen through this program are anticipated to advance and reach a larger patient population over time.

I-Care facilitated improved utilization of available services from the clinical medical model as demonstrated by increased continuity of care with primary care and engagement with the clinical pharmacy team, as well as decreased ED utilization and hospitalization. This suggests care needs were being met in the ambulatory setting, and that patients experienced decreased disease severity and/or improved control of comorbidities; an overall cost savings for the health system and the patient. Observed reductions in the mean HbA1c, LDL, and urine microalbumin demonstrate meaningful clinical change. The mean body mass index for the cohort showed minimal change, which could be attributed, in part, to a number of contextual factors. For instance, strict social distancing in the context of the COVID-19 pandemic has shown an adverse impact on individuals' eating behaviors and dietary habits, and food insecurity could also impact the quality of accessible food.^24,25^ It is also important to note that the COVID-19 pandemic limited the number of in-person clinic visits,^26,27^ which might have affected visits for the annual dilated eye examination.

The R-HLAT facilitated individualized, person-centered care by spurring real-time identification and addressing of knowledge gaps, and it also facilitated system-level efforts to improve individualized care. For example, medication management was identified as a substantial health literacy challenge within this cohort and, to address this challenge, I-Care incited individualized care coordination with the care manager and increased pharmacy engagement (scheduled visits, assessment of medication adherence, prior authorization support, and arrangement of Dispill prepackaged pills). Visits with the clinical pharmacist and nutritionist provided verbal and visual educational tools in patients' respective languages.

The involvement of culturally and linguistically congruent health navigators played an integral role at the intersection of health care and community. Health navigators engaged individually with patients who welcomed and consented to health navigator involvement in their care. This layer of trust and regular connection to health care and services allowed for ongoing educational reinforcement, follow up of care plans, and further attention to address the SDOH. Half of I-Care enrolled patients had no formal education, many of them were on public assistance or SSI for income support, and all, but one participant, were served by managed Medicaid or Medicare insurance. Health navigators and care managers assisted participants in engaging support services that were previously poorly understood and underutilized by this patient population.

The refugee and immigrant population faced with a high burden of social determinants impacting health equity described in this clinical program is similar to other urban academic medicine clinics in U.S. cities that resettle refugees.^28^ The generalizability of our findings may be limited by a relatively small number of patients followed here, but clearly demonstrates that improving continuity of care, engaging a multidisciplinary team, and building bridges to extend care beyond the walls of the clinic with care managers and health navigators from refugee backgrounds can improve outcomes. Future research will involve systematic evaluation of patients, providers, health outcomes, and cost analysis to move toward greater institutionalization of a care model that has demonstrated improved markers of health equity.

## Conclusion

The I-Care program emphasizes the importance of a multidisciplinary care model that incorporates a care manager for coordination between a clinical team and community health navigators to improve health equity in chronic disease management for refugee and immigrant patients. Adapting care models to engage with culturally and linguistically congruent community partners is essential to building trust and confidence, while improving care for a diverse patient population with disproportionate burden of social determinants.

## Abbreviations Used

BP: blood pressure
CBOs: community-based organizations
CCOC: Catholic Charities of Onondaga County
CDCES: Certified Diabetes Care and Education Specialist
CVD: cardiovascular
EHR: electronic health record
NY: New York
LDL: low-density lipoprotein
PCPs: primary care providers
RD: registered dietician
R-HLAT: Refugee Health Literacy Assessment Tool
RISE: Refugee and Immigrant Self-Empowerment
SDOH: social determinants of health
SUNY: State University of New York
